# Textile Supplies for Hospitals

**Published:** 1916-05-27

**Authors:** 


### TEXTILE SUPPLIES FOR HOSPITALS.

### XII.—The Purchasing of Stores.

The first step towards obtaining the maximum value for money lies in making a judicious selection from the goods best suited by their nature for the allotted work. What to buy is a problem most often settled by experience, direct or indirect, and if its dictates are not perfectly uniform the reason is that its criteria are rough. The experience of one is not quite the experience of another, either because of some imperfectly appreciated feature in the goods or in their treatment after purchase. But, in the main, experience leads aright even when its subsidiary processes are obscure, and the consensus of experience affords a solid foundation of which purchasers are quick to avail themselves. Every hospital acquires its individual experience, limited, it may be, by its own method of operations and coloured by its own surroundings, but still of extreme value to itself. Experience accumulates also amidst those who make a business of the supply of textiles to hospitals, and are in the way of receiving accounts from different sources. In any event, it is not difficult to assemble a collection of samples all warrantable to return fair satisfaction at their price and to answer every reasonable requirement, and it is not impossible to scrutinise their features and to see wherein the difference of value essentially resides.

### Methods of Buying.

The question of how to buy is almost entirely governed by the size of the orders that can be placed, and that of what to buy is not free from the same limitation. An institution purchasing upon a sufficient scale has an inducement to prescribe its own standards. Like a Government department, it may communicate the general nature of its wishes to a selected number of manufacturers, with a request for samples complying as fully as possible with the desiderata. These samples can then be tested for weight, purity, strength, elasticity, and fastness of colour, and the best of them can be used as a basis for a call for tenders. The arrangement assumes that the orders to be given are of an importance to make special exertions worth while on the part of manufacturers of the particular class of goods, and of a size also to make the production economical. The minimum quantity for manufacture to a precise specification varies with each class of article, and the larger the individual order can be made, the better from the point of view of economy in working.

It is beyond the facilities of a hospital to examine the most intimate nature of woven goods, and test and tabulate their characteristics. The work calls for experience and for a special equipment which can be most easily found in the public laboratories of the textile trade. The Testing House of the Manchester Chamber of Commerce, and a municipal institute, the Bradford Conditioning House, have the requisite facilities for examining and recording the essential features of textile goods and for drafting standard specifications to which deliveries of goods under contract should conform. In preceding articles of this series specimen specifications have been quoted, and in buying in accordance with precise tests the hospitals do no more than is done already by the Government Services, some public and a certain number of large private undertakings. The method may not be the most productive of a lively competition for the available business, but it does ensure the maintenance of quality and the supervision of points not appreciable by the unassisted senses of sight and touch. The desirability of having searching tests arises out of the ease with which superficial appearances may be made misleading. Granted that the manufacturer employs the same materials and same methods, his productions will be of a fairly consistent average of strength and quality from year to year. In this manner, and without systematic tests, the quality of standard cloths is maintained regularly, and to the satisfaction of all concerned. The test does no more than corroborate the reality of the pains expended in preserving the integrity of standard goods. But textiles are eminently goods which may be made to look the same without, in fact, being the same. They are articles fluctuating in cost, and in order to avoid changes of price, the quality can in many cases be degraded without obvious effect to the eye. While there are well-known makes which do not vary appreciably at any time, there are imitations of them of which the real inferiority can hardly be appreciated in the absence of the direct evidence of tests conducted with instruments of precision, and tests are above all necessary to those who throw open the gate to all comers.

### Direct and Indirect Purchase.

The guarantees of laboratory certificates are not the only ones available. The assurances of trusted dealers are of use, and there are trade-marked articles with the quality of which a manufacturer is forbidden by self-interest to tamper. Only in the articles in largest consumption by the largest institutions is there reason to look for a substantial saving in buying at the fountain-head. The matter is one of the size of orders, which must be large if the manufacturer is to produce cheaply. The middleman of established connection places large orders at favourable moments, keeps up a stock, and sells in broken quantities, and in charging a living profit for such services, adds nothing in reality to the cost of the goods. Were the manufacturer to proceed in the same way, he would of necessity have to ask similar prices. The skilled distributor sifts the suitable from the unsuitable, and he presents a greater variety of assorted articles than is ordinarily possible to the individual manufacturer.

Having arrived at a standard there is everything

Note.—Previous articles in this series appeared on December 18, 1915, and January 8, 22, February 5, 19, March 4, 18, April 1, 15, 29, and May 13, 1916. The next article will treat of "The Care of Textile Goods."

to be said in favour of cleaving to it until strong cause is shown for a change. To buy first one type and then another without sufficient experience of either, is to introduce disorder and confusion into the store department without compensating advantage. The results are untidiness and uncertainty in the wards, and haphazard purchasing can rarely be economical. The major benefit is got in buying the right article in such quantities as will ensure the lowest price and the most painstaking attention. Customers who obtain the name of being barely worth serving, come to be badly served, when with a better management of the distribution of their orders they could secure the ready co-operation of their suppliers. It is better from both points of view to give or receive one good order than a succession of small ones, and in buying seldom and in fair quantities the purchaser has a clearer view of what he is doing. When the quantities required can be estimated for some months ahead, there are frequently opportunities of saving by contracting at once for a series of future deliveries. The system is in force in all departments of textile industry, and from the hospital point of view it enables advantage to be taken of a favourable price without loss of interest or the encumbrance of a surplus stock.

### Supplies and Suppliers.

In dealing with manufacturers it is needful to turn to the primary markets; to Manchester for cotton sheets, quilts, prints, and the like; to Belfast and the East of Scotland for linen sheets and towels; to Yorkshire, Lancashire, and Witney for blankets; Rochdale and Wales for flannels; and to Bradford for uniform serges. The specialised distributors of hospital textiles are centred in London, and there are local retailers of drapery throughout the country. Local supplies may be good or less good, but attention can be drawn to one fact concerning them. There are fashions of place just as there are fashions of season. Conforming to local prejudice, local tradesmen buy and sell the only sorts of goods for which there is a local demand, and it is a fact easily verifiable in removing from one point of the country to another that the articles best known and liked in one district are unknown in some other area. It follows that local stock does not necessarily supply the most suitable article even for purely utilitarian purposes. In the great centres wider selections of available goods are to be found.

The larger part of woven textiles are manufactured anonymously, and reach the consumer by a somewhat devious course. Thus goods woven in the outskirts of Lancashire may pass to a quasi-manufacturer in Manchester and from him to a wholesale dealer in London, whose customers are retailers in different parts of the country through whom the public are supplied. The producer is diffident about letting his customer know of precisely what materials his goods are made, for the reason that the knowledge could be used against him. He prefers, therefore, to match a pattern supplied rather than to work to particulars narrowly laid down. In order not to forfeit his Manchester custom he abstains usually from doing direct business with London, and the system has to him at least the advantage of providing large orders and prompt payment for deliveries. Increasingly, the larger manufacturers are asserting their independence of localities, cultivating direct relations and the use of trade-marks identifying their own production. These marks become steadily better known, and in so far as they guarantee the quality of the cloths upon which they appear, they are of distinct use to purchasers.

In turn, these trade-marked goods afford a tempting target for imitation in similar-looking goods at an appreciably lower price. One effect is to make the proprietary article look the dearer, but it is not upon bare appearances that comparison should be made. Even in the absence of trade-marks and of the magic of advertising, the standard product of a great firm may be expected to look superficially dearer than goods produced under less weight of responsibility.

### Contract Forms.

Considerations of prudence require that in buying by tender the conditions of the contract should be clearly set forth, and more than one form of contract is in current use. By way of concrete example, one form may be given to show the matters to which importance is attached:—

*Conditions of Contract.*

1. The cost must include the price of delivery at . . .
2. The materials shall conform in all respects to the patterns and specification, and shall be subject to expert examination. Such examination will be at the expense of . . . , provided that the goods are in accordance with the standard patterns and specification. In the event of the goods not being up to standard, this cost will be debited to the contractor, and . . . reserve the right to take what action they may think fit in the matter.
3. All articles are to be of the best workmanship, the cloths being evenly woven with yarns of uniform thickness and regularity. Each piece to be marked with the actual length contained.
4. Invoices for goods to be subject to a cash discount of 2½ per cent., paid within one month of delivery.
5. . . . do not bind themselves to accept the lowest or any tender, nor will they undertake to assign any reason for the rejection of a tender. They further reserve the right to accept any tender wholly or in part.

The conditions are to be read in conjunction with the standard specification drawn up to meet the particular needs, and it will be seen that they refer to the cost of an expert examination of the delivered goods. This cost may be in the form of fees amounting usually to a few shillings, or be levied upon the value of the goods. In the latter case the examination may be expected to cost 1 per cent.; an addition to the net cost which may be viewed as a modest premium for insurance.

